# Design and Parameter Identification for a Positioning Platform with a Large Stroke and High Precision for Segmented Mirrors

**DOI:** 10.3390/mi14040713

**Published:** 2023-03-23

**Authors:** Zihao Yin, Rongjie Qin, Haoting Du, Weiyinuo Zhou, Jialin Sun, Dexin Sun, Yinnian Liu

**Affiliations:** 1 Key Laboratory of Infrared System Detection and Imaging Technologies, Shanghai Institute of Technical Physics, Chinese Academy of Sciences, Shanghai 200083, China; 2 University of Chinese Academy of Sciences, Beijing 100049, China

**Keywords:** active optics, 3-DOF parallel positioning platform, flexile amplification mechanism, particle swarm optimization, large stroke and high precision

## Abstract

An active optical system with three segmented mirrors was proposed to verify the co-focus and co-phase progress. In this system, a kind of large-stroke and high-precision parallel positioning platform was specially developed to help support the mirrors and reduce the error between them, which can move in three degrees of freedom out of plane. The positioning platform was composed of three flexible legs and three capacitive displacement sensors. For the flexible leg, a kind of forward-type amplification mechanism was specially designed to amplify the displacement of the piezoelectric actuator. The output stroke of the flexible leg was no less than 220 μm and the step resolution was up to 10 nm. Further, a linear model was established to identify the amplification ratio between the actuator and the flexible leg, which can increase the precision of the positioning platform. Moreover, three capacitive displacement sensors with a resolution of 2.5 nm were symmetrically installed in the platform to accurately measure the position and attitude of the platform. To improve the stability and precision of the platform, particle swarm optimization algorithm was applied to identify the control matrix, which can help the platform achieve ultra-high precision positioning. The results showed that the theoretical matrix parameters had a maximum deviation of 5.67% from the experimental ones. Finally, abundant experiments verified the excellent and stable performance of the platform. The results proved that while bearing the heavy mirror, which is no more than 5 kg, the platform can realize a 220 μm translation stroke and 2.0 mrad deflection stroke, with a high step resolution of 20 nm and 0.19 μrad. These indicators can perfectly cater to the requirements of the proposed segmented mirror system’s co-focus and co-phase adjustment progress.

## 1. Introduction

Research on large-aperture mosaic telescopes is an important aspect in the field of active optics [1]. Geostationary orbit imaging payloads with a large-aperture mosaic telescope can play a key role in the real-time detection of the Earth’s surface anomalies. However, the optical performance of this kind of telescope can only be achieved by a real-time co-focus and co-phase positioning adjustment for the segmented mirrors [2,3]. W.M. Keck [4] is a successful segmented ground-based telescope with three special hydraulic multistage actuators installed behind each mirror. To achieve a positioning adjustment of three degrees of freedom (3−DOF), the Thirty Meter Telescope (TMT) [5,6], which is in development, adopts three “soft” voice coil actuators and an active support structure similar to Keck. On 25 December 2021, NASA successfully launched the James Webb Telescope [7,8], which was unfolded in orbit and was composed of 18 segmented mirrors. There are seven low-temperature actuators, customized by Ball Aerospace, behind each mirror. Each actuator has a coarse and fine two-stage adjustment system, and the actuator is connected to the mirror and support mechanism through a kind of flexible structure [9,10]. The co-phase adjustment of most ground- or space-based telescopes depends on the positioning mechanism behind the mirror. For deployable segmented telescopes, an ultra-precision positioning system and complex control strategy are the key points of co-focus and co-phase adjustments. Research indicates that the segmented mirrors are expected to have excellent co-phase performance with piston errors below λ/20, and tip/tilt error below 0.5 μrad—here, λ is the wavelength of light [11,12]. To verify different co-focus and co-phase adjustment strategies, it is necessary to improve the precision of different positioning systems.

According to the inverse piezoelectric effect [13], piezoelectric ceramics can generate force and displacement, meaning that the special material has a strong load-bearing capacity and a high positioning accuracy. Combined with a flexible amplification mechanism [14], it can be used to develop a positioning platform to verify the co-focus and co-phase adjustment of the mirrors. Today, there are many studies focused on the multi−DOF piezoelectric positioning system. Hyeong-Geon Kim et al. [15] designed a 3−DOF positioning platform with multiple groups of amplification mechanisms superimposed as legs, achieving a tracking error of no more than 1 mrad in X− and Y−rotation. Hwa Soo Kim et al. [16] developed a miniature parallel 3−DOF platform for a high-frequency active vibration isolation system. Peng-Zhi Li et al. [17] manufactured a kind of nano positioner with the flexure diaphragm guider, capacitive sensors, and walking piezoelectric actuators to assist with biomedical technology and nano assembly. Zhi Li and Jinjun Shan [18] applied three piezo-actuators to tune the gap spacing between two optical band of an imaging Fabry−Perot spectrometer. Vahid Hassani and Tegoeh Tjahjowidodo [19] proposed a pyramidal-shaped 3−DOF piezo-driven mechanism to create elliptical motion in 3−D planes. Shijing and Yingxiang Liu et.al. [20] developed a novel two-axis piezoelectric tilting mirror to assist the optical micromanipulation. A cross-shaped piezoelectric composite beam based on simple bonded type bimorphs was applied to their design, and the experiments revealed its features of fast response, high positioning accuracy, and feasible motion tracking capacity. They also used a flexible anti-shear shell to increase the stiffness and resonance frequency of the steering mirror, and abundant experiments proved the advantage of this design [21]. However, most systems did not add position and attitude measurement devices, and they could only achieve semi-closed-loop positioning control. Moreover, their payload capability was too small for segmented mirror adjustments.

In order to verify the co-focus and co-phase detection and adjustment strategy of the segmented mirror, we designed a 3−DOF piezoelectric positioning system with a large stroke and high precision. The positioning system was supported by three flexible amplification legs equipped with piezoelectric actuators; meanwhile, three capacitive displacement sensors serve as a high-precision attitude feedback measurement system. The platform can generate large-stroke and high-precision adjustment in the out-of-plane 3−DOF errors of segmented mirrors. In order to accomplish a high-precision closed-loop positioning adjustment, the amplification ratio of the flexible leg was experimentally measured and the control matrix of the platform was identified through the measurement system. Each indicator was better than the system requirement, which guaranteed the high-precision co-focus and co-phase adjustment of segmented mirrors.

The second section of this paper introduces the structure and design of the piezoelectric positioning system. The third section describes the system control matrix of the piezoelectric positioning system, and proposes a matrix identification method based on particle swarm optimization algorithm. The fourth section introduces the experiments to identify the amplification ratio function and control matrix, and finally, we obtain the stroke and resolution performance of the proposed platform. A summary is provided in the fifth section.

## 2. Piezoelectric Positioning System

**Figure 1:**
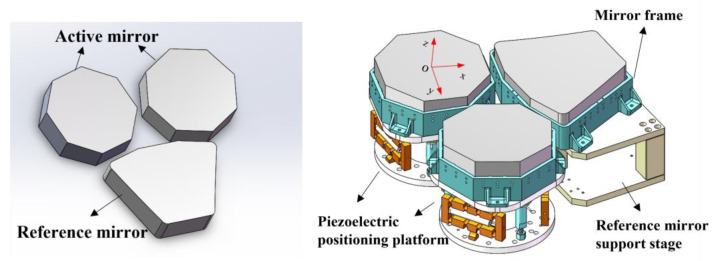
The active optical system with three spherical mirrors.

In an attempt to verify the real-time co-focus and co-phase adjustment for the segmented mirrors, a laboratory prototype with three spherical segmented mirrors was built, which was composed of an optical detection system and a piezoelectric positioning system. As illustrated in Figure 1, a fan-shaped mirror and two octagonal mirrors constitute a primary mirror module, and each mirror with a frame weighs no more than 5 kg. In this optical system, the fan-shaped mirror serves as a reference and the two octagonal active mirrors are supported by the piezoelectric positioning platforms. The platform assists the active mirror to adjust its position and attitude in 3-DOF (X−rotation, Y−rotation, Z−translation). It is worth mentioning that the errors in X−rotation, Y−rotation, Z−translation are described as tip, tilt, and piston in active optics, and the active optical system works at wavelength λ=600~900 nm. Therefore, the positioning platform needs to achieve a step resolution higher than 30 nm at least, and the X−rotation and Y−rotation resolution shall be higher than 0.5 μrad. To match our coarse positioning platform (a parallel 6−DOF position platform), the Z−translation stroke of the piezoelectric positioning platform shall not be less than 150 μm and the X/Y−rotation stroke shall not be less than 1 mrad. All indicators are listed in Table 1.

**Figure 2:**
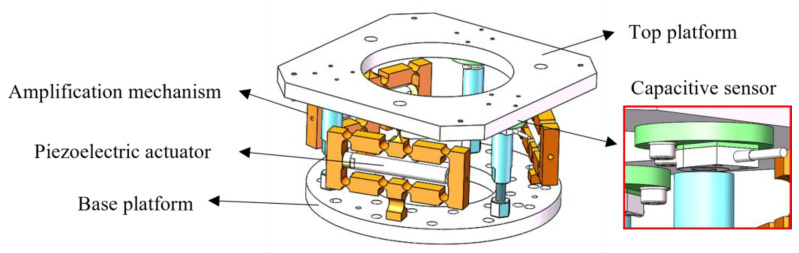
3−DOF piezoelectric positioning system.

The out-of-plane 3−DOF piezoelectric positioning system is mainly composed of a driving system and measurement system. As shown in Figure 2, three parallel flexible amplification mechanisms are linked with the top and base platforms by flexible hinges. Each flexible leg is driven by a piezoelectric actuator. In addition, three chip capacitive displacement sensors are uniformly installed on the back of the top platform. These sensors are used to measure the position and attitude of the top platform in 3−DOF.

### 2.1. Flexible Amplification Mechanism

**Figure 3:**
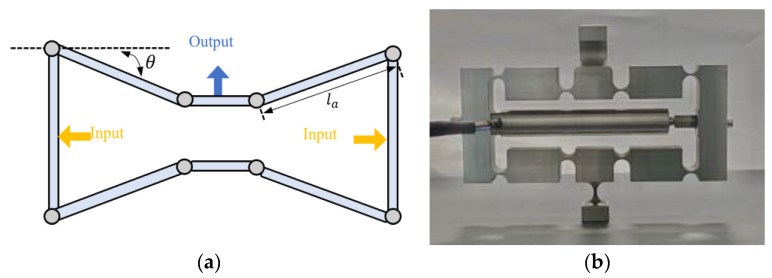
(**a**) Deformation of flexible amplification mechanism. (**b**) Flexible amplification mechanism with piezoelectric actuator.

A piezoelectric actuator generates displacement through an inverse piezoelectric effect, which has the advantages of strong bearing capacity and high displacement resolution. However, its stroke is extremely small—generally just tens of microns. In order to solve this problem, flexible amplification mechanisms are usually applied to increase the displacement stroke of the piezoelectric actuator. Significantly, flexible hinges with different rigidity and sizes can be designed to meet different kinds of load requirements, especially for space applications. Here, a kind of classical forward-type bridge amplification mechanism was adopted as the basic configuration. The amplification mechanisms designed in this study are shown in Figure 3. A flexible leg consisted of a circular flexible hinge, a spherical flexible hinge, and a flexible amplification mechanism. Additionally, the overall structure was centrosymmetric. Piezoelectric actuators were horizontally installed in the flexible amplification mechanism. The amplification ratio of the bridge amplification mechanism can be estimated using Equation (1) [22].

(1) $$R=\left[\sqrt{l_{a}{}^{2}sin^{2}\theta +\Delta x\left(2l_{a}cos\theta -\Delta x\right)}-l_{a}sin\theta \right]/\Delta x$$

As shown in Figure 3a, R is the amplification ratio of the mechanism, $l_{a}$ is the length of the diagonal arm, θ is the angle between the diagonal arm and the horizontal line, and ∆*x* is the input displacement. The optimized result of the diagonal arm $l_{a}$ is 25.55 mm, and the θ angle is 11.86°, while the amplification ratio is designed between 4.63 and 4.76. The parameters of the flexible amplification mechanism are usually optimized by considering stiff and inherent frequency [23,24,25].

Equation (1) is just a geometry-based estimation equation. Due to manufacturing and assembly error, the amplification ratio calculated by this equation is not accurate and the ratio changes according to the input force and deformation. In reality, the amplification ratio of the flexible mechanism is determined by the input displacement x of the actuator. Therefore, the relationship between the actuator and the amplification ratio can be written as Equation (2):

(2) $$d=R\left(x\right)*x$$

*R*(*x*) is the amplification ratio function of the flexible mechanism, *x* is the total displacement generated by actuator, *d* is the output of the flexible leg.

**Figure 4:**
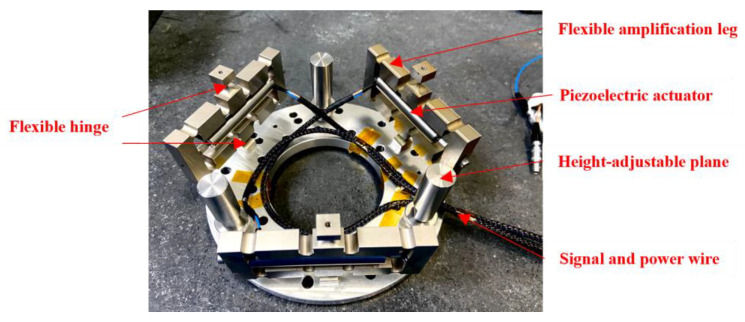
Three actuators assembled on the base platform.

As shown in Table 2, an actuator and a voltage amplifier from CoreMorrow were chosen. The maximum displacement of all selected actuators is no more than 60 μm. Meanwhile, their step resolution is up to 2 nm. Equipped with a flexible amplification mechanism, the legs can achieve a long stroke of no less than 220 μm. The strain gauge sensors (SGS) are used as feedback measuring devices in the actuators, whose closed-loop positioning accuracy is 5 nm. Figure 4 illustrates the actual assembly of three flexible amplification mechanisms.

### 2.2. Capacitive Displacement Sensor

**Figure 5:**
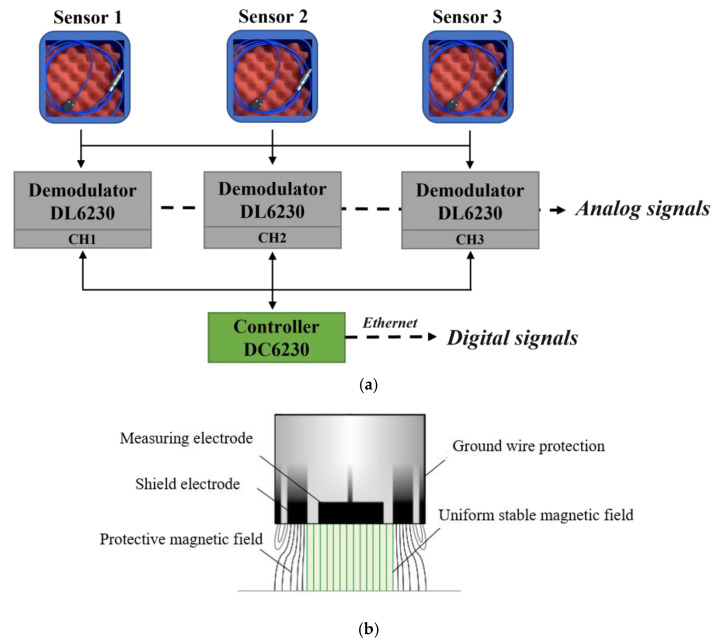
(**a**) Control system of capacitive displacement sensor. (**b**) Distance measuring diagram of capacitive sensor.

As shown in Figure 5a, three chip capacitive displacement sensors were used to measure the movement of the piezoelectric positioning platform. The capacitive displacement sensor is a kind of non-contact displacement sensor. The measured plane and the sensor both serve as a flat electrode. When providing the sensor with a constant and stable alternating current, the amplitude change in the alternating voltage is proportional to the distance between the sensor and the measured plane. By demodulating the alternating voltage, the output is converted to an analog signal.

**Figure 6:**
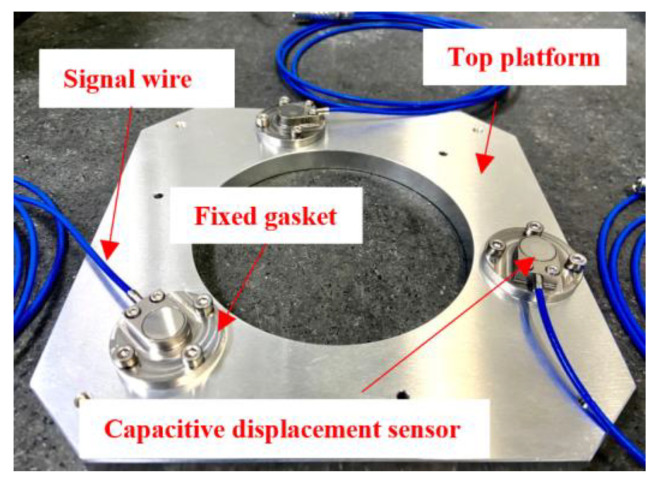
Three capacitive sensors assembled on the top platform.

As shown in Figure 6, in this piezoelectric positioning system, three capacitive displacement sensors were installed behind the moving platform to measure the 3−DOF position and attitude. The capacitive displacement sensors were produced by Micro−Epsilon company, and the main parameters are listed in Table 3. Combined with the controller DC6230 and signal processor DL6230 shown in Figure 5a, the standard maximum range of the sensor is no less than 1000 μm, and the measurement resolution is 2.5 nm.

With a high-precision measurement system and flexible amplification positioning system, the out-of-plane 3−DOF positioning platform can accomplish real-time positioning control with a large stroke and a high resolution. However, it is necessary to periodically identify and calibrate the system control matrix of the whole system to guarantee stability and high precision.

## 3. System Control Matrix and Identification Method

### 3.1. System Control Matrix

**Figure 7:**
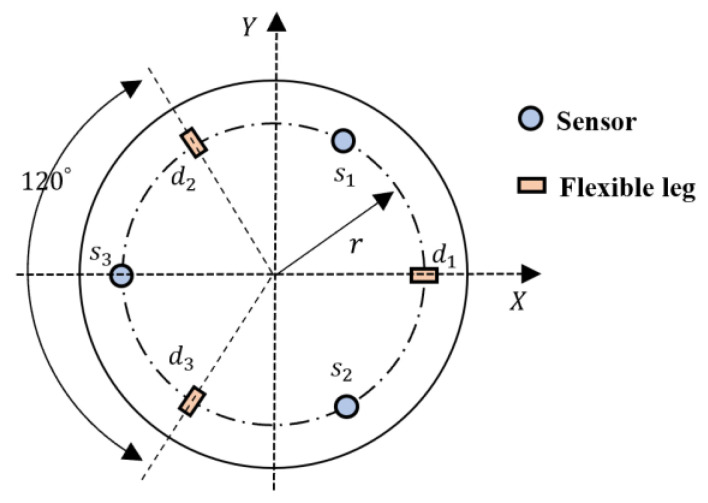
Geometric distribution of actuators and sensors.

As shown in Figure 7, the actuators and capacitive displacement sensors are symmetrically distributed on a circle with a radius of r, and the design value of r is 68.50 mm. The driving displacement generated by the flexible leg is $d_{i}\;\left(i=1,2,3\right)$, and the measured value of the capacitive displacement sensor is $s_{i}\;\left(i=1,2,3\right)$. The changes in the 3−DOF are z,α, β, where z is the translation of the platform along Z−axis, α is the rotation angle of the platform around the X−axis, and β is the rotation angle of the platform around the Y−axis.

According to the geometry, the kinematic relationship between the input displacement of flexible legs $d_{i}\;\left(i=1,2,3\right)$ and the output position and attitude of the platform z,α, β can be obtained.

(3) $$\left\{\begin{array}{c} z=\frac{d_{1}+d_{2}+d_{3}}{3}\\ \sin\alpha =\frac{-d_{2}+d_{3}}{\sqrt{3}r}\\ \sin\beta =\frac{-2d_{1}+d_{2}+d_{3}}{3r} \end{array}\right.$$

Since the tilt angle is very small, sinα ≈ α, sinβ ≈ β. Therefore, using Equation (3), the kinematic relationship is expressed in matrix form:

(4) $$\left[\begin{array}{c} z\\ \alpha \\ \beta \end{array}\right]\approx A\left[\begin{array}{c} d_{1}\\ d_{2}\\ d_{3} \end{array}\right]$$

where A is the driving matrix:

$$A=\left[\begin{array}{ccc} \frac{1}{3} & \frac{1}{3} & \frac{1}{3}\\ 0 & -\frac{1}{\sqrt{3}r} & \frac{1}{\sqrt{3}r}\\ -\frac{2}{3r} & \frac{1}{3r} & \frac{1}{3r} \end{array}\right]$$

The reverse input–output relationship of ${\left[\begin{array}{ccc} z & \alpha & \beta \end{array}\right]}^{T}$ and ${\left[\begin{array}{ccc} d_{1} & d_{2} & d_{3} \end{array}\right]}^{T}$ is:

(5) $$\left[\begin{array}{c} d_{1}\\ d_{2}\\ d_{3} \end{array}\right]\approx A^{-1}\left[\begin{array}{c} z\\ \alpha \\ \beta \end{array}\right]$$

Similarly, according to the measuring results of the capacitive displacement sensor ${\left[\begin{array}{ccc} s_{1} & s_{2} & s_{3} \end{array}\right]}^{T}$, the changes in the 3−DOF of the moving platform can be calculated. Then, the platform position and attitude can be measured according to Equation (6).

(6) $$\left[\begin{array}{c} z\\ \alpha \\ \beta \end{array}\right]\approx S\left[\begin{array}{c} s_{1}\\ s_{2}\\ s_{3} \end{array}\right]$$

The S matrix is called the measuring matrix.

$$S=\left[\begin{array}{ccc} \frac{1}{3} & \frac{1}{3} & \frac{1}{3}\\ -\frac{1}{\sqrt{3}r} & \frac{1}{\sqrt{3}r} & 0\\ -\frac{1}{3r} & -\frac{1}{3r} & \frac{2}{3r} \end{array}\right]$$

Additionally, the active control matrix between the input ${\left[\begin{array}{ccc} d_{1} & d_{2} & d_{3} \end{array}\right]}^{T}$ and the output ${\left[\begin{array}{ccc} s_{1} & s_{2} & s_{3} \end{array}\right]}^{T}$ is certain, as expressed in Equation (7).

(7) $$\left[\begin{array}{c} s_{1}\\ s_{2}\\ s_{3} \end{array}\right]=S^{-1}\left[\begin{array}{c} z\\ \alpha \\ \beta \end{array}\right]=S^{-1}A\left[\begin{array}{c} d_{1}\\ d_{2}\\ d_{3} \end{array}\right]$$

Let $C=S^{-1}A$, and the design value of this matrix can be calculated as:

$$C=\left[\begin{array}{ccc} c_{11} & c_{12} & c_{13}\\ c_{21} & c_{22} & c_{23}\\ c_{31} & c_{32} & c_{33} \end{array}\right]=\left[\begin{array}{ccc} \frac{2}{3} & \frac{2}{3} & -\frac{1}{3}\\ \frac{2}{3} & -\frac{1}{3} & \frac{2}{3}\\ -\frac{1}{3} & \frac{2}{3} & \frac{2}{3} \end{array}\right]$$

According to the characteristics of the platform, the active control matrix can be decomposed into three independent multivariate linear equations as Equation (8).

(8) $$\left\{\begin{array}{c} s_{1}=c_{11}d_{1}+c_{12}d_{2}+c_{13}d_{3}\\ s_{2}=c_{11}d_{1}+c_{12}d_{2}+c_{13}d_{3}\\ s_{3}=c_{11}d_{1}+c_{12}d_{2}+c_{13}d_{3} \end{array}\right.$$

**Figure 8:**
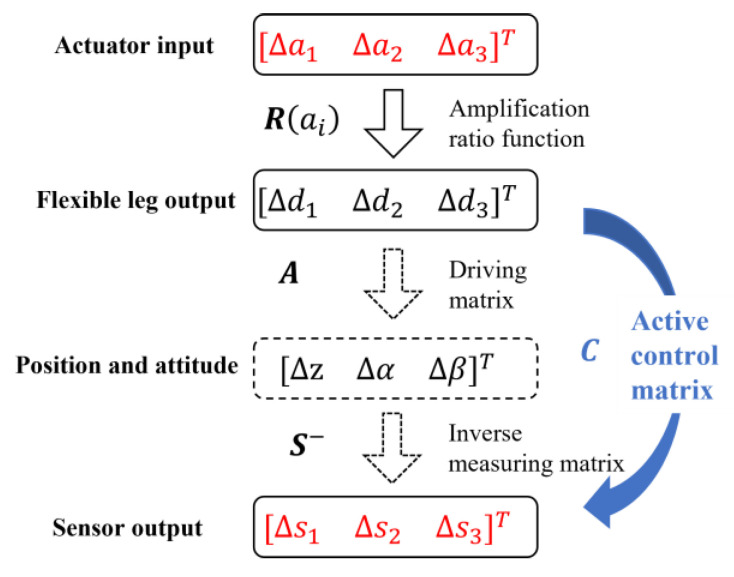
The relationship between actuator input and sensor output.

Finally, we obtained the output of the flexible legs as $d_{1}\;$=$\;R_{1}\left(a_{1}\right)a_{1}$, $d_{2}\;$=$\;R_{2}\left(a_{2}\right)a_{2}$, $d_{3}\;$=$\;R_{3}\left(a_{3}\right)a_{3}$ by substituting the identified amplification ratio function. Then, the relationship between the actuators and the sensors can be rewritten as Equation (9). Additionally, the matrix relationship between the input of the actuator and the output of the sensors is shown in Figure 8.

(9) $$\left\{\begin{array}{c} s_{1}=c_{11}R_{1}\left(a_{1}\right)a_{1}+c_{12}R_{2}\left(a_{2}\right)a_{2}+c_{13}R_{3}\left(a_{3}\right)a_{3}\\ s_{2}=c_{11}R_{1}\left(a_{1}\right)a_{1}+c_{12}R_{2}\left(a_{2}\right)a_{2}+c_{13}R_{3}\left(a_{3}\right)a_{3}\\ s_{3}=c_{11}R_{1}\left(a_{1}\right)a_{1}+c_{12}R_{2}\left(a_{2}\right)a_{2}+c_{13}R_{3}\left(a_{3}\right)a_{3} \end{array}\right.$$

Here, $R_{i}\left(a_{i}\right)$ is the amplification ratio of the No *i*. flexible leg when the corresponding displacement of actuator *i* is $a_{i}$ (*i* = 1,2,3).

Due to manufacturing tolerance and misalignment error, the real control matrix of the positioning platform is different from the design value. It is necessary to periodically calibrate the active control matrix to improve the positioning accuracy of the system and satisfy the requirements of co-focus and co-phase adjustments.

### 3.2. PSO Method for Identification

Particle swarm optimization (PSO) is a heuristic evolutionary computation method, which originates from the study of the behavior of birds’ predation [26]. The fundamental idea of a particle swarm optimization algorithm is to search for the optimal solution through cooperation and information shared among individuals in a large population. With two attributes, speed and position, each particle represents a candidate solution to the problem. Speed represents the speed and direction of particle movement, and position represents the iterative process of the candidate solution. Each particle separately searches for the optimal solution in the search space and marks it as the extreme value of the current individual $P_{best}$. Then, each share the $P_{best}$ with others to find the best value: the global optimum $G_{best}$. At each iteration, all particles update their speed and position by their $P_{best}$ and the global optimum $G_{best}$. The updated equations are as follows:

(10) $$pos_{i}{}^{k+1}=pos_{i}{}^{k}+v_{i}{}^{k+1}$$

(11) $$v_{i}{}^{k+1}=\omega \times v_{i}{}^{k}+c_{1}\times rand\times \left(P^{k}{}_{best,i}-x_{i}{}^{k}\right)+c_{2}\times rand\times \left(G^{k}{}_{best,i}-x_{i}{}^{k}\right)$$

where ω is the inertia coefficient (the larger the *ω*, the better the global search performance), and $c_{1}\;c_{2}$ are learning factors. rand is a random number range from 0 to 1. $P^{k}{}_{best,i}$ is the best value of particle *i* in the *k*th iteration, while $G^{k}{}_{best}$ is the global best value in the *k*th iteration.

The fitness value calculated by the fitness function is a standard used by the algorithm to determine whether the population has approached the optimal solution. Equation (12) gives the designed fitness function for solving the control matrix.

(12) $$fitness=\frac{\sum_{i=1}^{n}{\left(a_{1}d_{ij}+a_{2}d_{ij}+a_{3}d_{ij}-s_{ij}\right)}^{2}}{n}\;\left(j=1,2,3\right)$$

where $\left[a_{1},a_{2},a_{3}\right]$ is the solution of each particle; n is the size of data; $d_{ij}$ is the output displacement of the jth flexible amplification mechanism in data group i; and $s_{ij}$ is the measured value of the jth capacitive displacement sensor in data group i.

The steps of solving control matrix parameters by a particle swarm optimization algorithm are as follows:

(1) Initialize the parameters of the particle swarm optimization algorithm (including learning factor, weight coefficient, particle swarm number, maximum number of iterations, accuracy index).
(2) In the extreme range, initialize the particle swarm position and speed, solve the particle fitness, and find the individual optimal value and the global optimal value.
(3) Update speed and position according to position equation and speed equation.
(4) Calculate the individual fitness value and update the individual optimal value and the overall optimal value.
(5) If the overall fitness reaches the target accuracy or the maximum number of iterations, stop searching and output the overall optimal value. Otherwise, skip back to step (3).

## 4. Experiments

### 4.1. Amplification Ratio Identification

**Figure 9:**
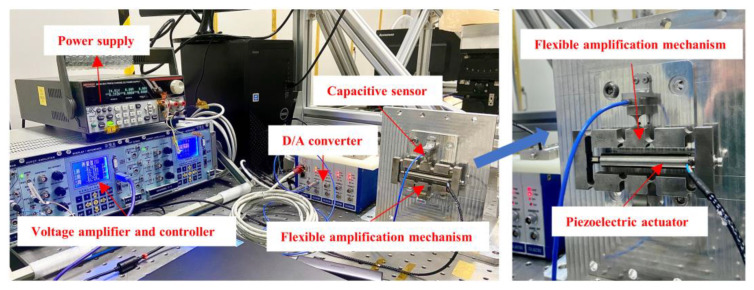
Experimental system of amplification ratio function.

As shown in Figure 9, the platform for testing the flexible amplification mechanism is constructed. The experimental platform is composed of three flexible legs, three capacitive displacement sensors, a three-channel signal A/D conversion system, a three-channel piezoelectric amplifier, and a computer control system. The whole experimental platform is firmly installed on the optical stabilization platform.

**Figure 10:**
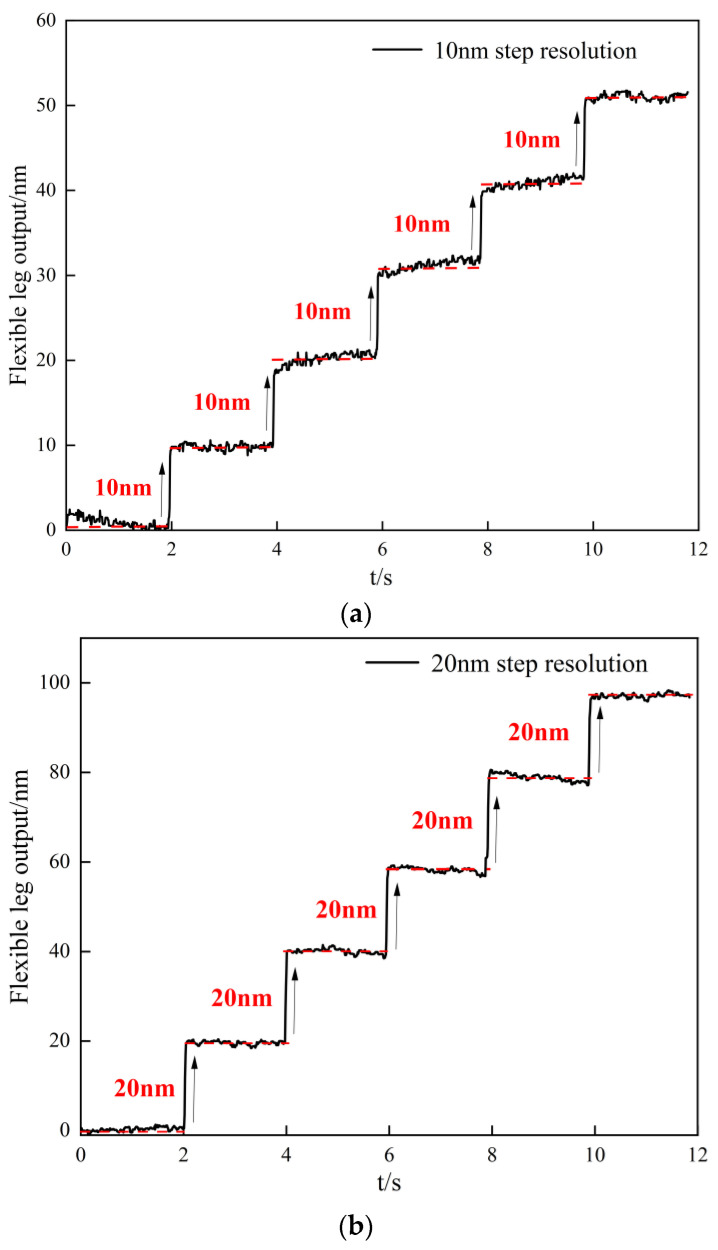
(**a**) Output resolution of the flexible leg with open-loop 5 mV voltage input (**b**) Output resolution of the flexible leg with SGS-closed-loop 5 nm displacement input.

By the experimental system in Figure 9, we test the open-loop resolution and the SGS-closed-loop resolution of the flexible legs. As for open-loop resolution, the step input to the piezoelectric actuator is a 5 mV voltage step which can drive actuators to generate a 2~3 nm step output. As shown in Figure 10a, by the amplification of the mechanism, the legs can achieve a 10 nm displacement. With regard to closed-loop resolution, the actuators’ output displacement is set as 5 nm according to the SGS’s accuracy. As shown in Figure 10b, the closed-loop resolution of the legs is stable at 20 nm.

Although the open-loop resolution of the flexible leg is higher than closed-loop performance, the closed-loop control is still important because it can reduce the hysteresis of the piezoelectric actuators.

For the open-loop control, we sent a triangle voltage wave (the peak-to-peak value of the volt is 100 V, and the frequency is 0.1 Hz) to the actuator. Next, we applied the corresponding displacement triangle wave input to the actuator by SGS-closed-loop control. The test results are shown in the following figure. The entire displacement output of the flexible leg is measured by the external capacitive displacement sensor. The hysteresis effect can be described as Equation (13).

(13) $$hy=\sqrt{\frac{\max\left({\left(y_{output}-y_{target}\right)}^{2}\right)}{\max\left(y_{target}{}^{2}\right)}}$$

Here, hy means an indicator to weigh the hysteresis. $y_{output}$ is the output displacement of the flexible leg. $y_{target}$ is the standard triangle wave.

**Figure 11:**
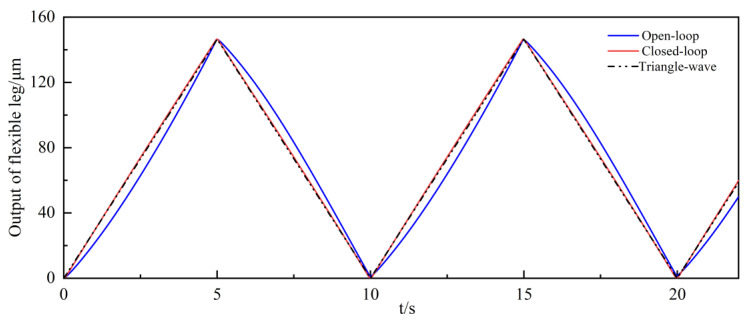
Hysteresis effect of the flexible leg with open-loop and closed-loop control.

As shown in Figure 11, the hysteresis for open-loop control is 7.13%, while the hysteresis of closed-loop is only 0.31%. The closed-loop control can significantly reduce the hysteresis of the piezoelectric actuator. This result shows that the SGS-based closed-loop control of the flexible leg is beneficial to the precise positioning performance of the positioning platform.

**Figure 12:**
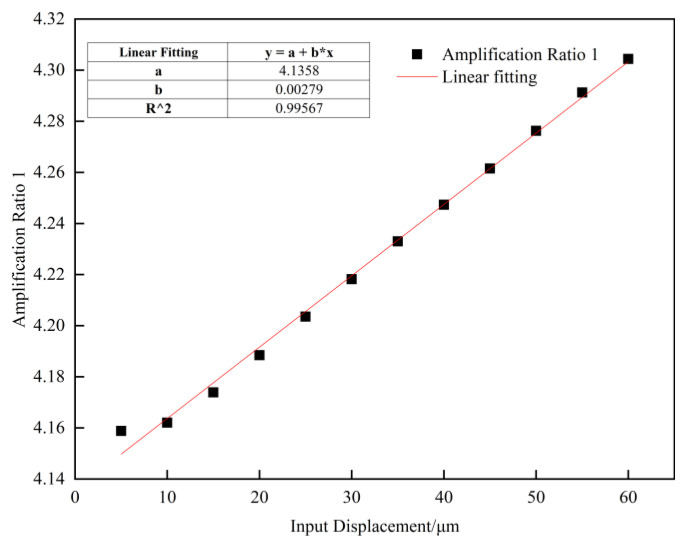

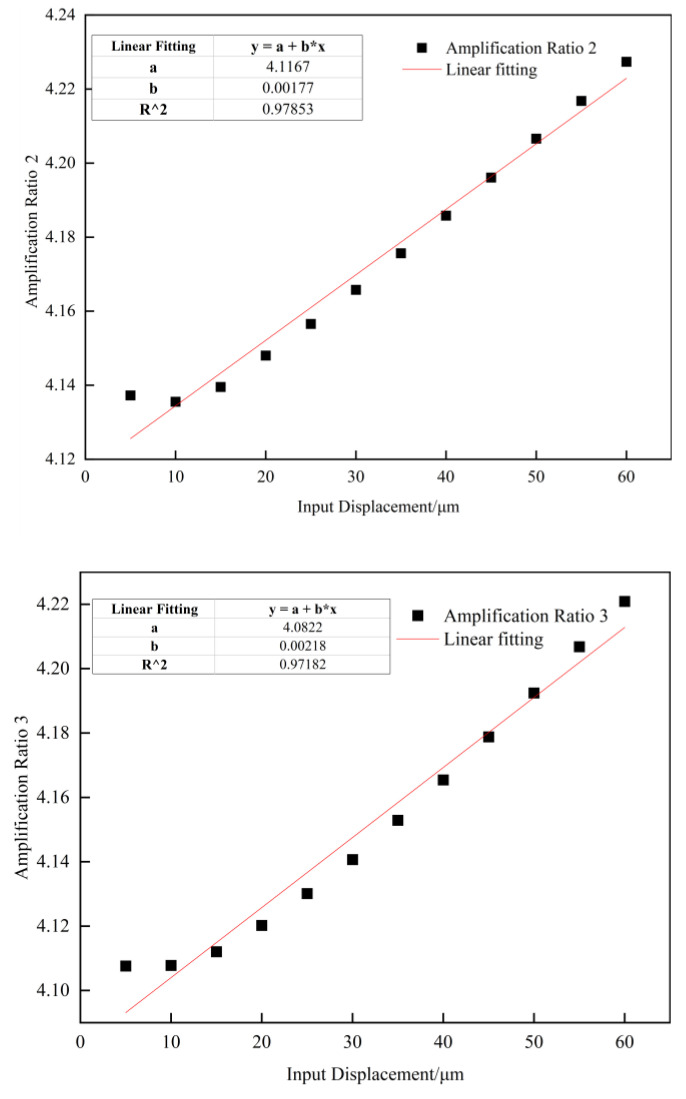
Curve fitting of amplification ratio function.

As for the amplification ratio of the flexible leg, we applied the SGS-based closed control to finish the experiments. Considering a 5 μm step displacement input of the piezoelectric actuator, the step displacement of the flexible amplification mechanism is measured by the capacitive displacement sensor. The relationship between the amplification ratio and the input displacement of the actuator can be obtained using the linear regression method, which is shown in Figure 12.

According to the experimental data, as the input displacement increases, the amplification ratio also increases slightly, and the overall variation range of the amplification ratio is within 5%. In general, it is smaller than the design amplification ratio range of 4.6–4.7 because Equation (1) used in the amplification design is estimated according to the geometric relationship, without considering the details of material properties and mechanical deformation. Therefore, the designed value is slightly different from the actual situation.

### 4.2. System Control Matrix Identification

**Figure 13:**
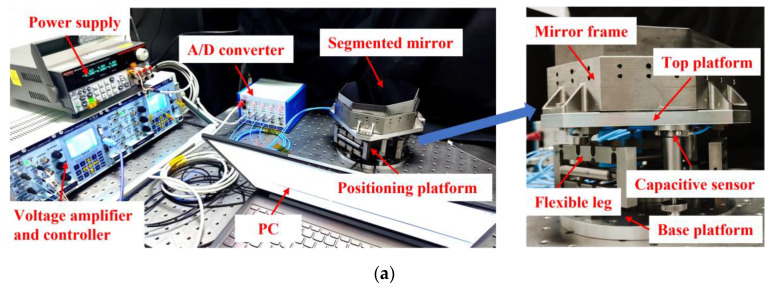

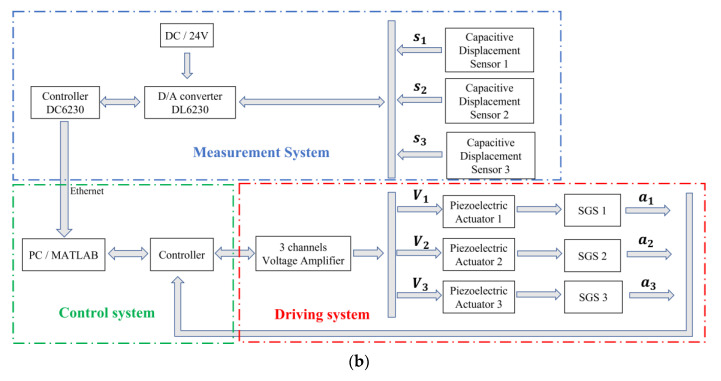
(**a**) Experimental system for 3−DOF piezoelectric positioning platform. (**b**) Schematic diagram of experimental system.

As is shown in Figure 13, the 3−DOF piezoelectric positioning platform is formed by a top platform, the capacitive displacement sensor, the flexible amplification legs, and a base platform. The overall testing system is presented in Figure 13a. The PC sends the voltage control command to the lower computer controller and acquires the measured value of the internal SGS of the actuator output using the lower computer controller, while collecting the measured value transmitted from the signal demodulator of the capacitive displacement sensor. All experiments are completed on the optical precision platform.

As shown in Figure 13b, within the stroke range of the actuator, three actuator displacement changes were randomly provided, and the measured values of the capacitive sensors were recorded. One hundred groups of data were obtained by repeating the experiment. Depending on the difficulty of the problem, the size of population is generally between 20 and 60, and the maximum number of iterations is generally no less than 50. More particles lead to a faster convergence but more calculations. The learning factor is usually set as 2. With the increase in iterations, the weight decreases, and the searching gradually changes the focus from global to local. The accuracy index is generally determined based on the calculation of fitness value. After a comprehensive consideration of efficiency and experimental test, the main parameters of PSO were determined and are shown in Table 4.

**Figure 14:**
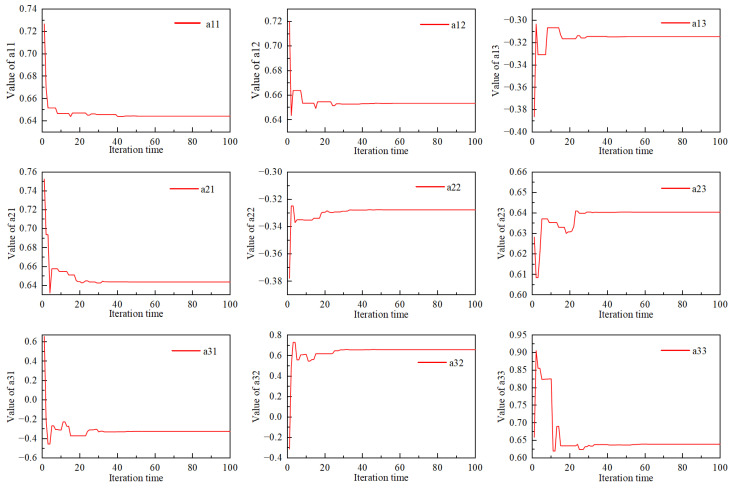
PSO convergence process for searching the matrix parameters.

Figure 14 shows the convergence process of each matrix parameter.

**Figure 15:**
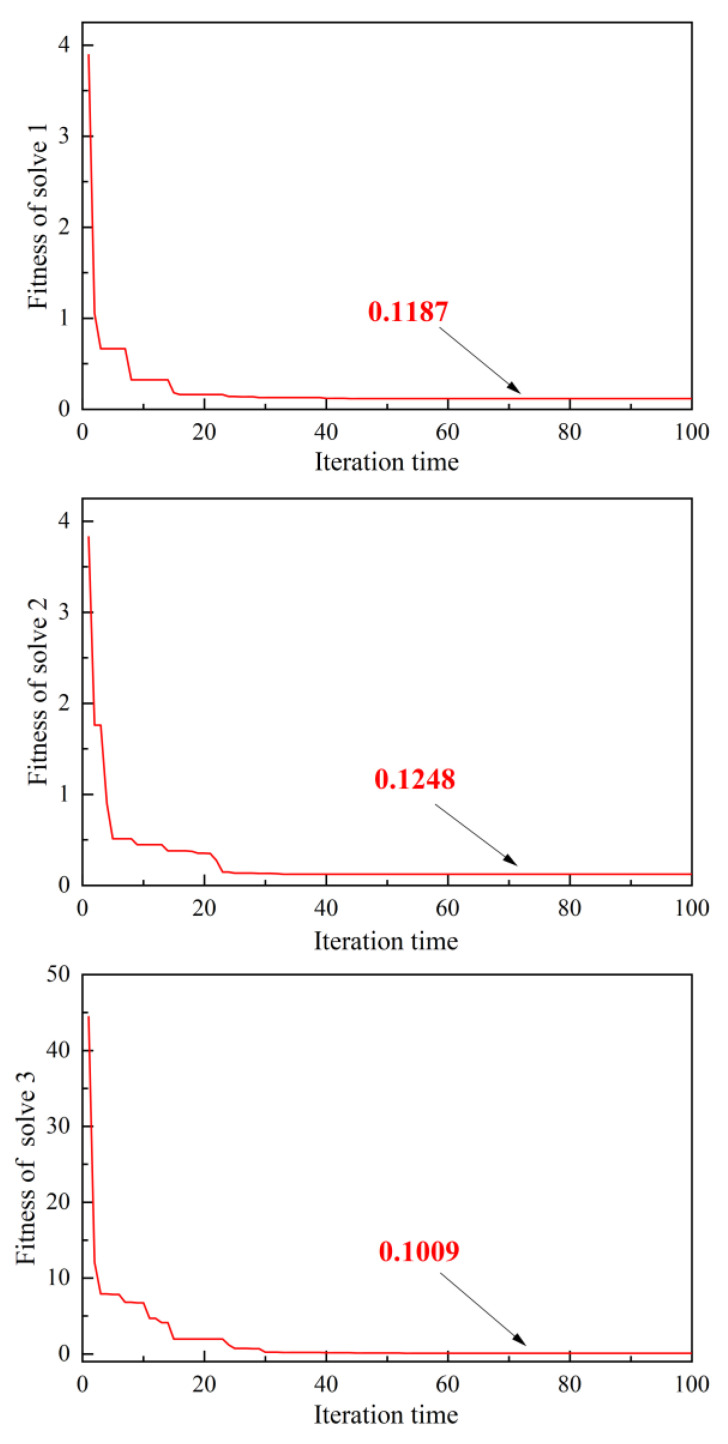
Fitness convergence progress of each solution.

Corresponding to three linear equations shown in Equation (9), the particle swarm optimization algorithm was run three times. The fitness convergence of each solution is illustrated in Figure 15.

The algorithm convergences fast and the particles in the population find the best optimum within 50 generations. Considering that the size of the population is 20, the algorithm runs efficiently. As shown in Figure 15, the final best fitness of the population is not zero. The reason is that a systematic error exists and the data gathered from the SGS and capacitive displacement sensor are limited by synchronization and accuracy.

Finally, the identified control matrix $C_{test}$ found by the particle swarm optimization algorithm is:

$$C_{test}=\left[\begin{array}{ccc} 0.644390739130132 & 0.653490547857818 & -0.314429230955206\\ 0.643928269914274 & -0.327588704100672 & 0.640511486455441\\ -0.323327449429491 & 0.660552700506762 & 0.639812798539691 \end{array}\right]$$

The maximum error percentage between the identified matrix and the design value is 5.67%. The error of the control matrix is mainly derived from the processing and installation error of the upper and lower platforms and the manufacturing error of the flexible amplification mechanism. This error can be significantly reduced by improving the manufacturing accuracy and adopting more precise assembly and adjustment methods.

### 4.3. Platform Performance Verification

Given the maximum displacement of the corresponding flexible amplification leg, the measured values of the corresponding capacitive displacement sensors were recorded and the maximum travel of the 3−DOF calculated. The maximum output displacement of three actuators is given at the same time, and the maximum travel of the Z−axis is measured. The No. 1 and No. 2 piezoelectric actuators hold still, and the No. 3 piezoelectric actuator outputs a maximum displacement; then, the maximum rotation around the X−axis is measured. Similarly, the No. 2 and No. 3 piezoelectric actuators do not move, and the No. 1 piezoelectric actuator generates a maximum displacement; then, the maximum rotation around the Y−axis can be measured.

As is shown in Table 5, the piezoelectric positioning platform can achieve no less than a 220 µm translation and no less than a 2 mrad rotation, which perfectly meet the large-stroke requirements of the segmented mirrors.

**Figure 16:**
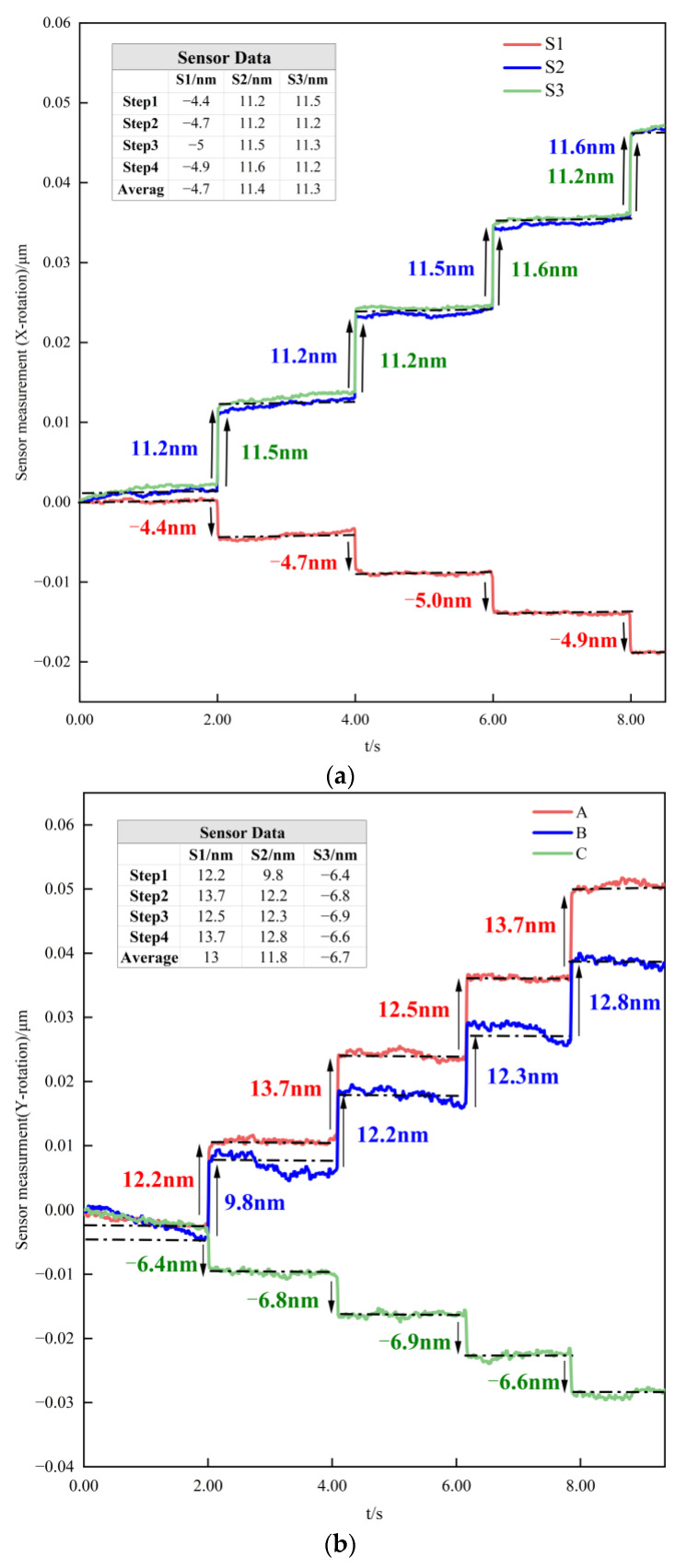

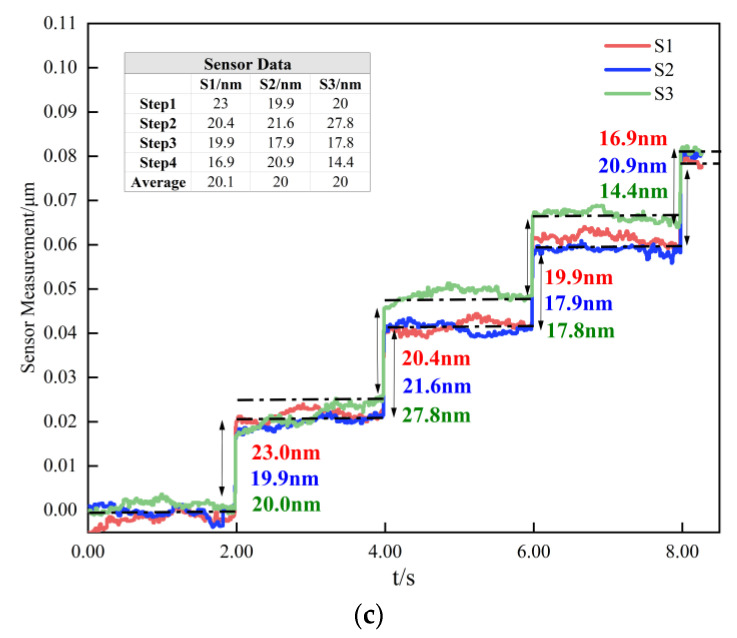
(**a**) Sensor measurement of minimum rotation around X−axis. (**b**) Sensor measurement of minimum rotation around Y−axis. (**c**) Sensor measurement of minimum translation by Z−axis.

Give the minimum displacement or voltage step input of the different actuators, the minimum displacement of the platform can be obtained by measuring the attitude change in the platform using a capacitive displacement sensor. The No. 1 and No. 2 piezoelectric actuators keep still, and the No. 3 piezoelectric actuator outputs a minimum displacement of 5 nm. Then, the minimum rotation of the X−axis can be solved by the measurement of three capacitive displacement sensors. Similarly, the No. 2 and No. 3 piezoelectric actuators do not move, and the No. 1 piezoelectric actuator generates the minimum displacement; then, the minimum rotation around the Y−axis can be detected by solving the measurement of sensors. If three actuators have a step of 5 nm at the same time, we can obtain the minimum displacement in the Z−axis. Here, to avoid the environment noise and improve the resolution of the capacitive displacement sensors, the sampling time of the sensors are set as 104.1 Hz. The results are shown in Figure 16.

Through the data shown in Figure 16 and the measurement matrix, the step resolution of the piezoelectric positioning platform can be calculated and is shown in Table 6. The platform realizes a minimum rotation of no more than 0.14 µrad around the X−axis and 0.19 μrad in Y−axis, and the minimum translation along the Z−axis is no more than 20 nm. The resolution performance can meet the requirement of the co-focus and co-phase adjustments of segmented mirrors.

## 5. Summary

It is known that the performance of an active optical system for segmented mirrors relies on the positioning system behind the mirrors. In order to reduce the piston, tip, and tilt error of each mirror, we developed a large-stroke and high-precision piezoelectric positioning platform based on three capacitive displacement sensors and three flexible amplification legs. We specially designed and optimized a kind of forward-type flexible amplification to amplify the stroke of the piezoelectric actuator. It is worth mentioning that we applied a closed-loop control based on the SGS data to linearize the output of the piezoelectric actuator. Moreover, because of manufacturing and assembly error, the actual performance of the amplification ratio was usually different from the design value. According to the analysis and experimental results, we treated the amplification ratio as a linear function and obtained the precise parameters of each flexible leg. The experiments showed that a single flexible leg could achieve a large stroke of no less than 220 μm, and the closed-loop step resolution was up to 20 nm. Then, to improve the stability and the precision of the positioning platform, the particle swarm optimization algorithm was introduced to identify the control matrix with a large amount of data acquired by the experiments. The optimized results showed that the practical control matrix had a maximum deviation of 5.67% from the designed ones. The accurate control matrix was important to improve the precision of the nano-positioning platform. Finally, the experiments showed that the piezoelectric positioning system can realize a translation by the Z−axis of no less than 220 µm and a rotation in the X/Y−axis of no less than 2 mrad, while the resolution along the Z−axis was higher than 20 nm, and the rotation resolution around the X− and Y−axis was higher than 0.19 µrad. All indicators of the positioning platform could meet the requirements of the real-time co-focus and co-phase adjustments of the proposed active optical system. This work is of good significance to future research on the co-focus and co-phase detection and adjustment strategy of segmented mirrors.

## Figures and Tables

**Table 1:** The indicators designed for the piezoelectric positioning platform.

Indicator	Value
Z−translation stroke	>150 μm
X−rotation stroke	>1 mrad
Y−rotation stroke	>1 mrad
Z−translation resolution	<30 nm
X−rotation resolution	<0.5 μrad
Y−rotation resolution	<0.5 μrad
Load weight	5 kg

**Table 2:** The parameters of the piezoelectric actuators and amplifier.

Items	Parameters	Items	Parameters
Actuator	PSt150/5/60 VS10 (CoreMorrow)	Voltage amplifier	E00.D6 (CoreMorrow)
Standard stroke	60 μm	Maximum output	150 V
Resolution	2 nm	Resolution	5 mV
Length	64 mm	Inner sensor	SGS
Rigidity	8 N/μm	Sensor accuracy	5 nm

**Table 3:** The main parameters of the capacitive displacement sensors.

Items	Parameters
Type of sensor	CSH1FL−CRm1,4 (Micro−Epsilon)
Standard measuring range	1000 μm
Reduced/extended measuring range	500 μm/2000 μm
Resolution static (DT6230) (2 Hz)	2.5 nm/5 nm/10 nm
Resolution dynamic (DT6230) (5 kHz)	25 nm/50 nm/100 nm

**Table 4:** Set parameters of the PSO algorithm.

Parameter	Value
Population M	20
Maximum iteration $K_{max}$	100
Learning factor $c_{1}$ $c_{2}$	2
Maximum weight $\omega_{max}$	0.8
Minimum weight $\omega_{min}$	0.2
Accuracy ε	0.5

**Table 5:** 3−DOF stroke of piezoelectric platform.

	No. 1 Sensor (µm)	No. 2 Sensor (µm)	No. 3 Sensor (µm)	Stroke (µm/mrad)
Z−axis translation	225.73	208.6	227.71	220.68 µm
X−axis rotation	−81.72	154.71	154.86	2.0 mrad
Y−axis rotation	157.52	159.28	−78.07	2.3 mrad

**Table 6:** 3−DOF minimum resolution of piezoelectric platform.

Position and Attitude	Resolution
Rotation in X−axis/Tip	0.14 µrad
Rotation in Y−axis /Tilt	0.19 µrad
Translation in Z−axis /Piston	20 nm

## Data Availability

Not applicable.
